# Aesthetic Values of the Buccal Fat Pad Excision in Middle-Aged Patients

**DOI:** 10.1093/asjof/ojac015

**Published:** 2022-03-04

**Authors:** Takayuki Kubo

**Affiliations:** Dr Kubo is a plastic surgeon in private practice, Tokyo, Japan

## Abstract

**Background:**

Despite buccal fat pad (BFP) excision being an effective and established surgery for the reduction of lower facial volume, there are very few reports explaining a systematic and reliable method for this surgery. The author will demonstrate one method for BFP excision surgery with some important tips and information on the procedure.

**Objectives:**

The author performed BFP excision surgery for patients seeking facial slimming or improvement of the ptotic lower face. To know the true efficacy of this surgery, objective facial scales were compared preoperatively and postoperatively.

**Methods:**

Preoperative and postoperative three-dimensional (3D) pictures were taken, and numerical data for cheek volume and horizontal lower facial length were obtained from 3D picture analysis. The data were statistically analyzed to reveal postoperative improvements.

**Results:**

The average age of the patients was 54.23 ± 10.31 years old. BFP excision surgery took 60.30 ± 5.93 minutes. Preoperative and 12 months postoperative body weight was 52.3 ± 6.84 and 52.3 ± 6.75 kg, respectively. The average weights for excised BFP were 1.74 ± 0.72 g right and 1.59 ± 0.71 g left. The average of preoperative lower facial volumes was 209.36 ± 30.22 mL right and 209.03 ± 30.13 mL left. The average 12 months postoperative lower facial volumes were 205.00 ± 30.37 mL right and 204.43 ± 30.02 mL left. The results showed a significant difference in both sides (*P* < 0.01). The average of horizontal lower facial lengths was 238.73 ± 10.87 mm preoperatively and 235.06 ± 10.57 mm 12 months postoperatively. The results affirm that there is a significant difference (*P* < 0.01).

**Conclusions:**

A properly executed BFP excision can achieve distinct facial slimming for the capacious face and can also contribute to improving lower face sagging due to BFP ptosis in middle-aged patients.

**Level of Evidence: 4:**

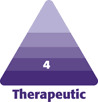

With the advent of modern medicine, our prolonged lifespan makes it possible for us to enjoy a better life. However, the emergence of an aged appearance is a concern for many people over time, making aesthetic surgeries increasingly popular. Facial ptosis is undeniably experienced by all ethnic groups, especially at both the periorbital region and the lower face. Although antiaging treatments for the periorbital region are well established,^1,2^ indications and methods for ptotic facial regions are highly diversified, and the selection of treatment should be differentiated depending on the specific condition and patient needs.

Patients as well as surgeons are always seeking out safer treatments for facial rejuvenation. For example, skin tightening therapies^3^ using ultrasound, radio frequency energy, and thread lifting methods for skin suspension are viable alternatives. Among those adjunctive facial tightening methods, thread lifting is frequently performed because of the simplicity of the procedure and minimal complications when using absorbable threads. The efficacy and long-term results, however, are not fully known and require further investigation.^4^

For patients seeking the appearance of distinct facial rejuvenation, the leading solution is facelift surgery^5^ involving the removal of excess skin and tightening of the superficial musculoaponeurotic system. It should be noted, however, that a facelift involves certain risks^6,7^ such as scars at incision lines or temporary hypesthesia. Due to these considerations, facelift surgery is not necessarily suitable for everyone. This has led the author to propose that excision of the buccal fat pad (BFP) is a legitimate alternative to facelift surgery, as it can deliver similar moderate facial slimming without the associated complications.

BFP excision originated in Europe in the eighteenth century.^8^ Traditionally, this procedure has been selected for conditions such as lip lacerations or traumatic facial defects in reconstructive surgery.^9^ For aesthetic purposes, the main candidates for BFP excision were young patients with prominent lower cheeks, a condition known as “chipmunk cheeks.” To some extent, a sagging lower face is also attributed to BFP ptosis. While there are very few papers documenting the value of BFP excision for ptotic lower face, the author theorizes that treatment with optimal results is BFP management.

Through treating many Asian patients in daily practice, the author came to realize that the Asian population has a relatively larger volume of BFP than other ethnicities. As they reach middle-age, their BFP begins to droop, resulting in the characteristic lower cheek sagging. The author has performed the BFP excisions, especially for middle-aged patients with ptotic lower cheeks, for whom a conventional facelift may have been indicated. This paper discusses the efficacy of BFP excision not only for the capacious face but also for middle-aged ptotic lower cheeks.

## METHODS

This study follows the Declaration of Helsinki and its guiding principles. Thirty patients (28 females and 2 males), age range 32-71 visited our clinic for the treatment of capacious or ptotic lower cheeks during the period March 2016 through December 2021, all of whom are included in this study. Candidates choosing BFP excision over facelift surgery tend to be those with a relatively capacious face, especially those with northern Asian background where face volume reduction can be more beneficial than skin tightening through a facelift, and those seeking only minor improvements while prioritizing the scarless surgery of BFP excisions from inside the mouth. One surgeon performed the entire process involving 12 months of postoperative follow-up. One female patient separated from this study group was followed up to 3 years postoperatively. Contraindications to this surgery are patients with previous complete BFP excision, those with injectable materials that can cause chronic inflammation in BFP, patients predisposed to severe bleeding, and where there are severe restrictions in mouth opening. Therefore, examination excluded patients with bleeding diathesis, remarkable jaw restrictions, and patients with previous BFP excisions. However, where clear residue was present from previous BFP surgery, revision surgery was performed conservatively so as not to injure surrounding tissues. For patients prescribed antithrombotic drugs, intake was withdrawn 5 days before the day of surgery. The examination was carried out in the seated position to evaluate bilateral BFP volumetric difference. BFP excision surgery was performed only in patients with objective BFP protrusion, or in patients with ptotic cheek of the anterior-medial portion of the face, rather than posterior-lateral, where the *masseter* muscle is located.

Informed consent was received, and all associated risks were explained before the surgery. Both plain and three-dimensional (3D) pictures (manufactured by the Morpheus Co. Ltd., Yongin, Gyeonggi, South Korea) were taken preoperatively and postoperatively at each patient’s visit to the clinic. The 3D pictures were taken in a controlled environment with each patient sitting at a fixed position in the room, with matched lighting, distance, and seating height.

**Figure 1.**
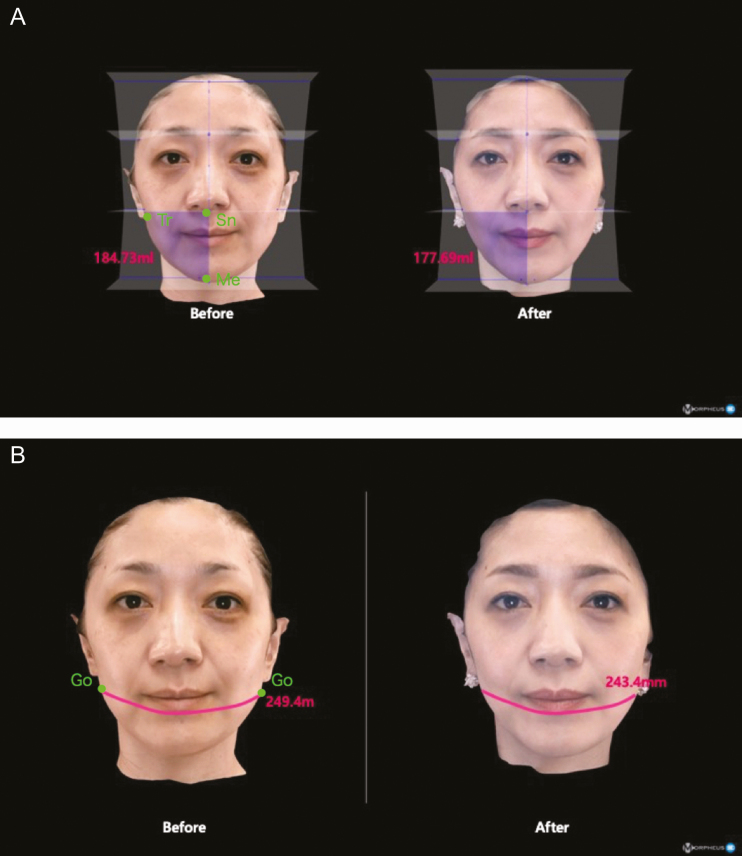
Comparison between preoperative and postoperative facial volume and lateral lower facial length. Facial volume analysis was performed using facial landmarks of the tragus (Tr), subnasal (Sn), and menton (Me). (A) Facial landmarks are indicated as green dots. The Morpheus 3D software (Morpheus Co., Ltd, Yongin, Gyeonggi, South Korea) automatically analyzed bilateral lower facial volume surrounded by those facial landmarks. This is a typical patient showing a significant capacity loss of the right cheek after the buccal fat pad excision. (B) The distance between skin surface above bilateral gonion (Go) indicated as green dots is measured for lateral lower facial length preoperatively and postoperatively. In this patient, there was a clear reduction in lateral lower facial length postoperatively.

Twelve months postoperative images were compared with preoperative images to identify reduced facial volume and size. Facial landmarks were placed on the skin surface as Gonion (Go) for bilateral jaw salient point positions, Menton (Me) for center of the Mentum, Subanale (Sn) for below the nasal tip, and Tragus (Tr) for lower margin of the ear lobes, respectively. Based on those facial landmarks, bilateral cheek volume was measured at the area surrounded by Tr, Sn, and Me (Figure 1A). And the horizontal lower facial length was measured at the distance between the bulging skin surface due to BFP above bilateral Go preoperatively, and flatter skin surface postoperatively. (Figure 1B). Bilateral BFPs collected during surgeries were weighed respectively.

Measurements and analysis were performed by the author using the Morpheus software with the help of a technician at the Morpheus Co., Ltd (Yongin, Gyeonggi, South Korea). The data were statistically analyzed to determine any significant differences preoperatively and postoperatively. The excised bilateral BFP weight difference was statistically compared using the Mann-Whitney test. Volumetric difference preoperatively and postoperatively was analyzed using the Wilcoxon test. The horizontal lower facial length was analyzed using the corresponding *t* test. Data were also examined to find any correlation between excised BFP weight and facial parameters. Correlations between extirpated BFP weight and horizontal lower facial length and volumetric change were statistically analyzed using the Pearson product-moment coefficient test. Preoperative and 12 months postoperative body weight was measured with differences analyzed using the Wilcoxon test.

### Surgical Technique

Before starting surgery, the oral cavity is examined for missing and/or artificial teeth. Detachable artificial teeth are removed, while orthodontic appliances are left in their original positions. Patients with temporomandibular disorder or trismus are asked to inform the surgeon in advance, in order to determine the full range of mouth opening. After establishing an intravenous line, the patient is placed in a supine position. Mild sedation using diazepam (10~20 mg) and hydroxyzine hydrochloride (12.5~25 mg) is introduced to relax the patient and to prevent hypertension, which may bring excessive intraoperative and postoperative bleeding. Throughout the surgery, blood pressure and other vital signs are closely monitored. After disinfecting the oral cavity with 7% Iodine Gargle solution, the mouth is kept open by placing a plastic wedge-shaped spacer between the back molars on the opposite side of the treatment area.

**Figure 2.**
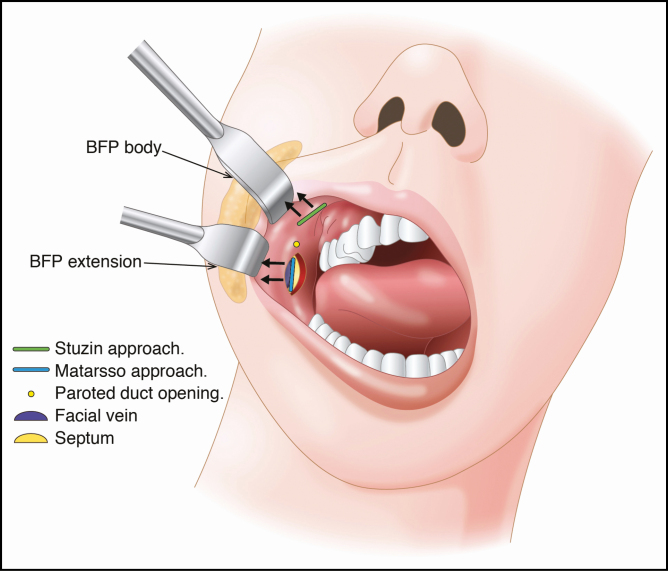
The schematic entry point of buccal fat pad excision at the right side. A green line illustrates the Stuzin approach. The black arrows at the Stuzin approach shown in this figure are directed upward to the buccal fat pad (BFP) body which is located at the superior cheek. This part of BFP is the first encounter from this approach. A blue line shows the Matarasso approach. Black arrows are directed transversely, reaching the BFP extension located at the lower cheek from this approach. With the Matarasso approach, approximately 1 cm straight incision is made longitudinally from 1 cm inferior-laterally of the parotid duct opening (yellow circle), and the buccinator muscle is dissected to reach the BFP septum (yellow semicircle). The facial vein (purple semicircle) is frequently encountered superior to the BFP septum.

With the mouth open, the parotid gland opening on the oral vestibule, situated obliquely below the upper second molar, is precisely located as a landmark point. The patient is locally anesthetized using an injection of approximately 6 mL of 1% xylocaine with 1:100,000 epinephrine into each oral vestibule. Then a longitudinal lateral incision is made, approximately 15 mm, using an radiofrequency (RF) electrode probe in caudal direction from 1 cm below the parotid gland opening on each oral vestibule. This is called the Matarasso approach^10^ as shown in Figure 2. If bleeding points develop while dissecting the buccinators, it is electro-coagulated with the bipolar hemostatic forceps. Then, blunt dissection is carried out using bipolar forceps until the buccinator is dislodged deep to the BFP capsule (septum), through which the yellowish BFP profile will become visible.

During this procedure, the facial vein branch is identified, running along BFP anterosuperiorly (Figure 2). The BFP space is always located posterior-medial to the facial vein, and these veins can be a useful landmark when the BFP position is not easy to identify or locate. In some patients, the facial vein branch may be engorged due to compression by a local anesthetic injected into the BFP pocket. In this patient, the BFP capsule should be dissected with great care to avoid injury to the facial vein. The area around the BFP capsule has numerous capillaries that easily bleed, so the distal end of the capsule should be clamped and elevated with the forceps while being dissected. Whenever bleeding starts on the stumps, it needs to be stopped completely to keep the surgical area clean and clear. After repeating this procedure several times, the capsule gradually becomes thinner and BFP begins to well out into the mouth. The BFP portion that comes out through the Matarasso approach is the distal end of the extension branch. This should be resected adequately, as this portion is the main BFP branch causing capacious or ptotic cheek. The distal end of the BFP extension is then clamped with the forceps and pulled with a gentle maneuver and resected appropriately. After the adequate resection of BFP extension, the BFP body part is pulled succeedingly and resected discreetly to avoid over-resection. All other remaining parts of the BFP, namely the whole temporal and the pterygoid, are preserved intact. After these procedures are completed, the spacer placed between the back molars is removed. With the mouth closed, a gentle massage using the palm of the hand is applied in all directions to the facial surface. This external pressure on the cheek will uncover the remaining excessive BFP which should be excised. Compression in all directions is also given intraorally using a cotton pad held with the forceps. This procedure ensures that there is no hidden bleeding point that may cause silent bleeding after the operation.

**Figure 3.**
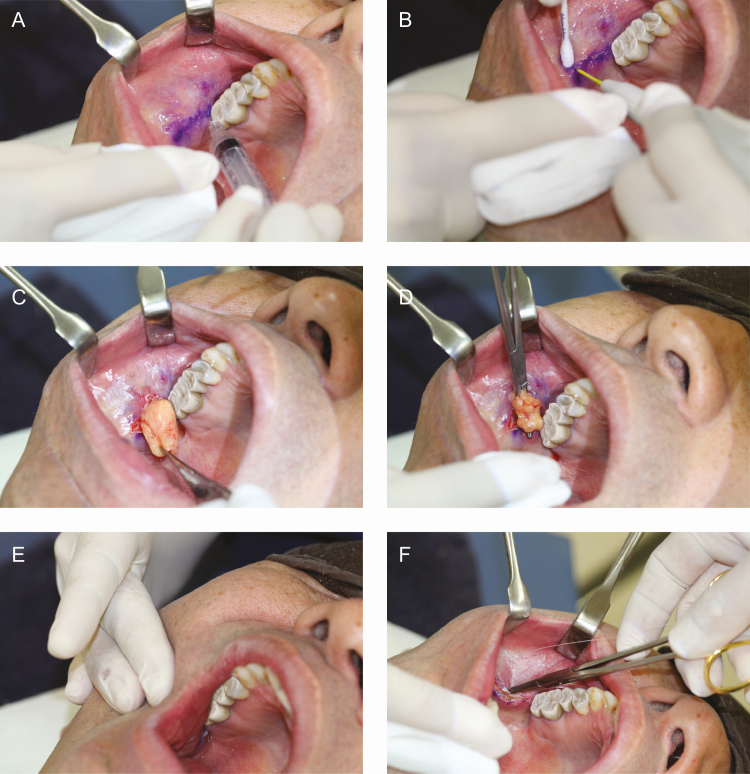
Series of pictures for buccal fat pad excision procedure taken for the right oral cavity from upward obliquely. After marking the incision line as shown in the purple marker, 6 mL anesthesia is infused into the buccal fat pad (BFP) pocket of a 58-year-old male patient. (B) A 1-cm longitudinal incision is made by RF electric blade, while the oral membrane is pressed by the Q-tip so that easier dissection is carried out. (C) After dissection down to the BFP septum, the BFP is automatically withdrawn with a help of small traction of forceps. (D) The tip of the BFP is pinched by forceps, then dissected piece by piece to avoid over-resection. (E) After approximate resection of the BFP, external pressure by the operator digits is made to verify if there is any residual fat. If so, residual BFP should be trimmed off carefully to not resect excessively. (F) Surgery is completed with a mucosal membrane stitch.

After dissecting and extirpating the BFP appropriately, the wound is closed with a 7-0 nylon suture. These are removed 7 days postoperatively. In cases where the patient was unable to attend the postoperative appointment to remove sutures, an absorbable suture was used. Sequential pictures of the BFP excision and its procedure are shown in Figure 3 and Video 1. The patient is kept in a reclining supine position, and the face is compressed with ice packs and a compression garment for at least 1 hour postoperatively. Before the patient returns home, complete hemostasis is reconfirmed, and the patient’s postoperative photographs are taken.

## RESULTS

In total, 30 patients (28 females and 2 males) of BFP excision were carried out by the author during the period from March 2016 to December 2021. The average age was 54.23 ± 10.31 (32-71) years. BFP excision surgery took 60.30 ± 5.93 minutes. Patients were followed and tracked postoperatively at intervals of 1, 3, 6, and 12 month(s). Follow-up typically ended after the 12-month appointment.

Nineteen patients had only BFP excision, and 11 patients had other procedures such as transconjunctival lower blepharoplasty (7 patients), upper blepharoplasty (3), and chin liposuction (1). Preoperative and 12 months postoperative body weights were 52.3 ± 6.84 and 52.3± 6.75 kg, respectively, and the body weight difference was not statistically significant (*P* = 0.74) (Table 1).

**Table 1.** Body Weight

Variable	Preoperatively	12 months postoperatively
Body weight	52.3 ± 6.84 kg	52.3 ± 6.75 kg

The average weights for excised BFP were 1.74 ± 0.72 g right and 1.59 ± 0.71 g left (Table 2). Excised fat weighed more for the right side in 21 patients and more for the left side in 9 patients. The excised bilateral BFP weight difference was statistically compared. Results showed no significant difference in bilateral extirpated BFP weight. The average of preoperative lower facial volumes was 209.36 ± 30.22 mL right and 209.03 ± 30.13 mL left. The average 12 months postoperative lower facial volume was 205.00 ± 30.37 mL right and 204.43 ± 30.02 mL left. Volumetric differences were statistically significant preoperatively and postoperatively on both sides (*P* < 0.01) (Table 3). The average of preoperative and 12 months postoperative horizontal lower facial lengths (Go distance) was measured in the distance between bilateral Go marked on the skin surface. The results were 238.73 ± 10.87 mm preoperatively and 235.06 ± 10.57 mm postoperatively. Results showed a significant difference preoperatively and postoperatively (*P* < 0.01) (Table 3). Correlations between extirpated BFP weight and volumetric change and horizontal lower facial length were statistically analyzed. There was no significant correlation in the relationship of each. All patients were questioned to rate the satisfaction of the result at 12 months postoperative follow-up, and this survey was conducted by a blind evaluator. The scale was ranged from 0 to 3 (0: poor, 1: no difference, 2: better, and 3: much better). Results showed 0 for 6.6%, 1 for 13.3%, 2 for 26.6%, and 3 for 53.5% fair. None of them expressed any major complaints.

**Table 2.** BFP Average Weight

Variable	Right	Left
Excised BFP weight	1.74 ± 0.72 (0.6-3.6) g	1.59 ± 0.71 (0.5-3.6) g

BFP, buccal fat pad.

**Table 3.** Cheek Volume and Length

Variable	Preoperatively	12 months postoperatively	*P* value
Right lower facial volume (mL)	209.36 ± 30.22	205.00 ± 30.37	<0.01
Left lower facial volume (mL)	209.03 ± 30.13	204.43 ± 30.02	<0.01
Go distance (mm)	238.73 ± 10.87	235.06 ± 10.57	<0.01

**Figure 4.**
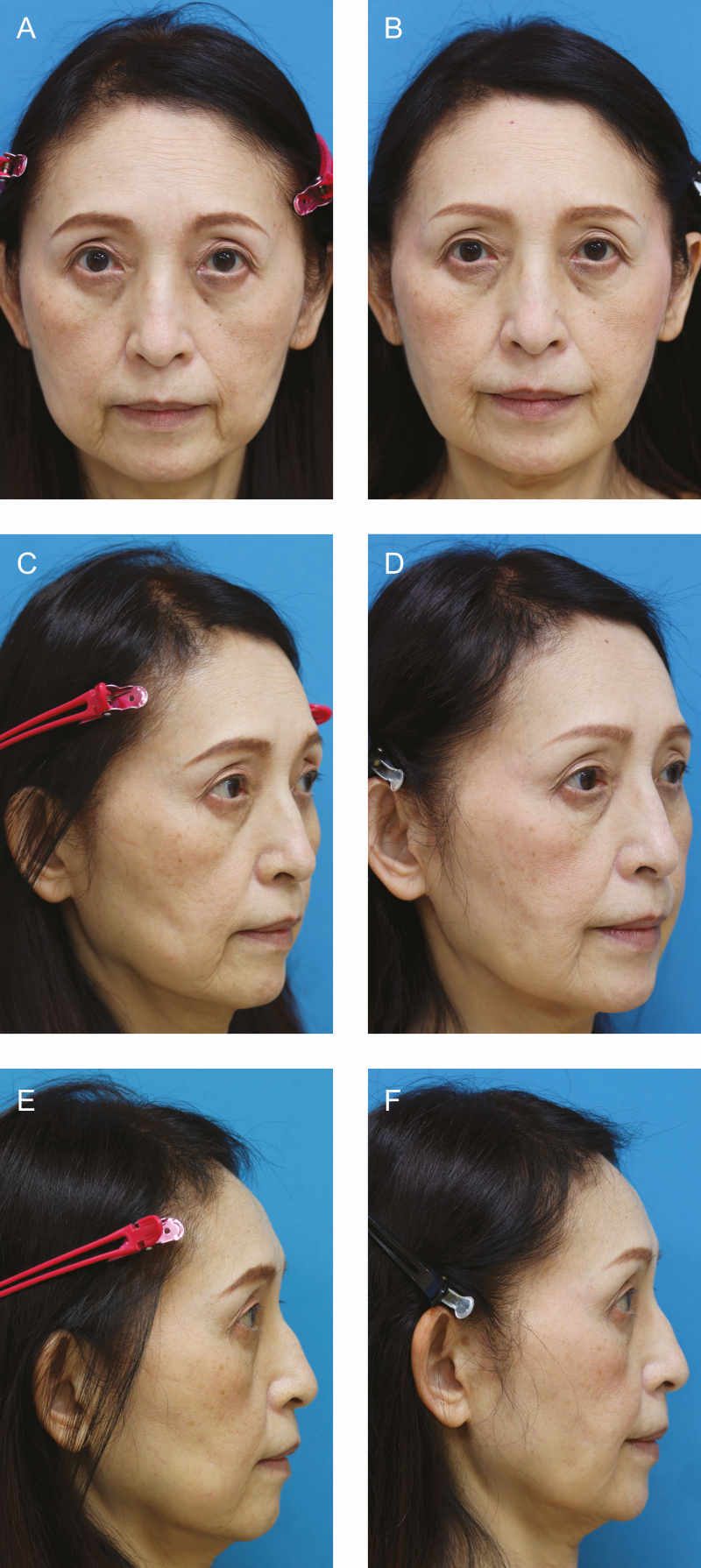
Patient 1: (A, C, E) Pseudo-herniation of the buccal fat pad was observed in this 62-year-old female. (B, D, F) Twelve-month postoperative photographs show that her lower facial volume was reduced, resulting in an improvement of slackening cheeks. The last picture was taken on December 22, 2021.

**Figure 5.**
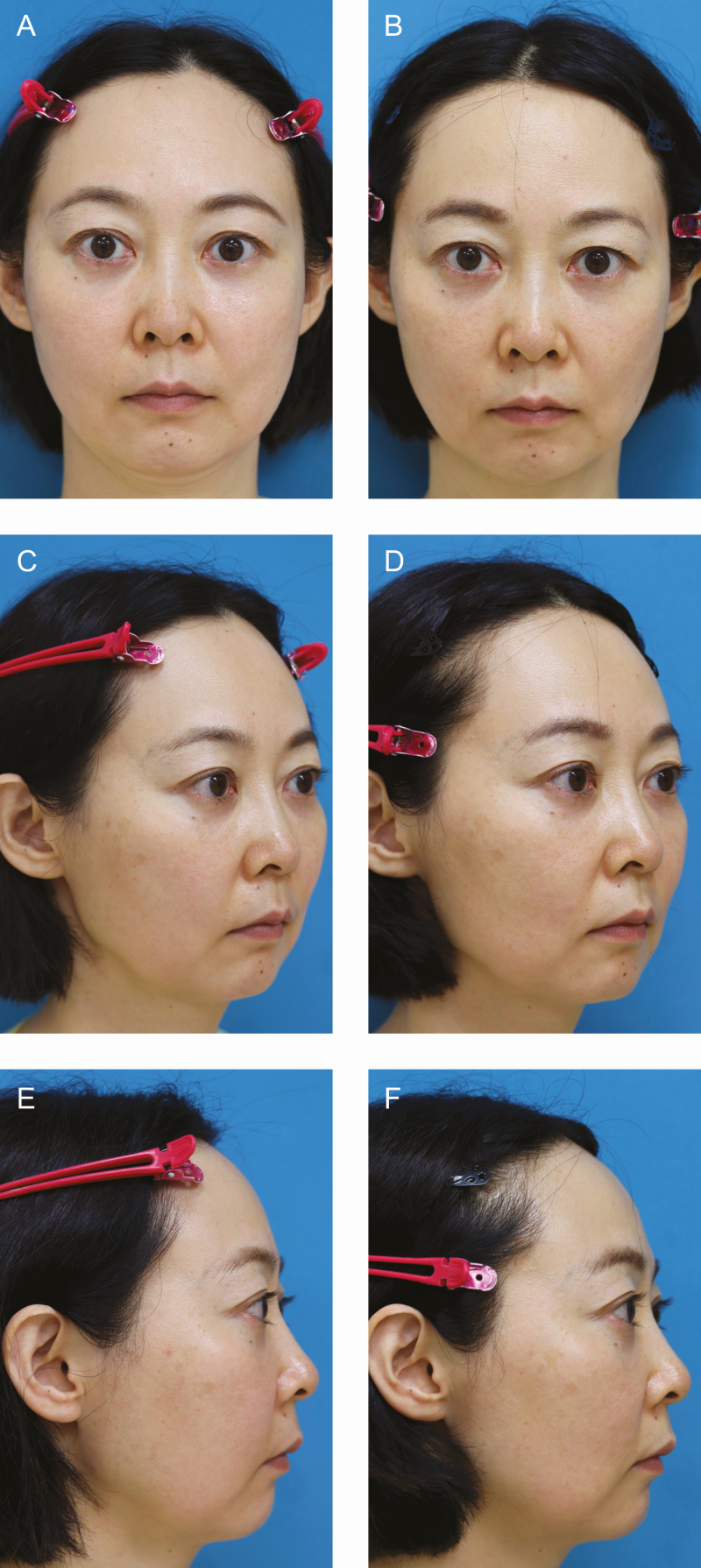
Patient 2: (A, C, E) Preoperative pictures of a 46-year-old female with typical round and capacious cheek. (B, D, F) Her facial contour became slimmer and pointed postoperatively. Twelve-month postoperative photographs were taken on September 22, 2021.

**Figure 6.**
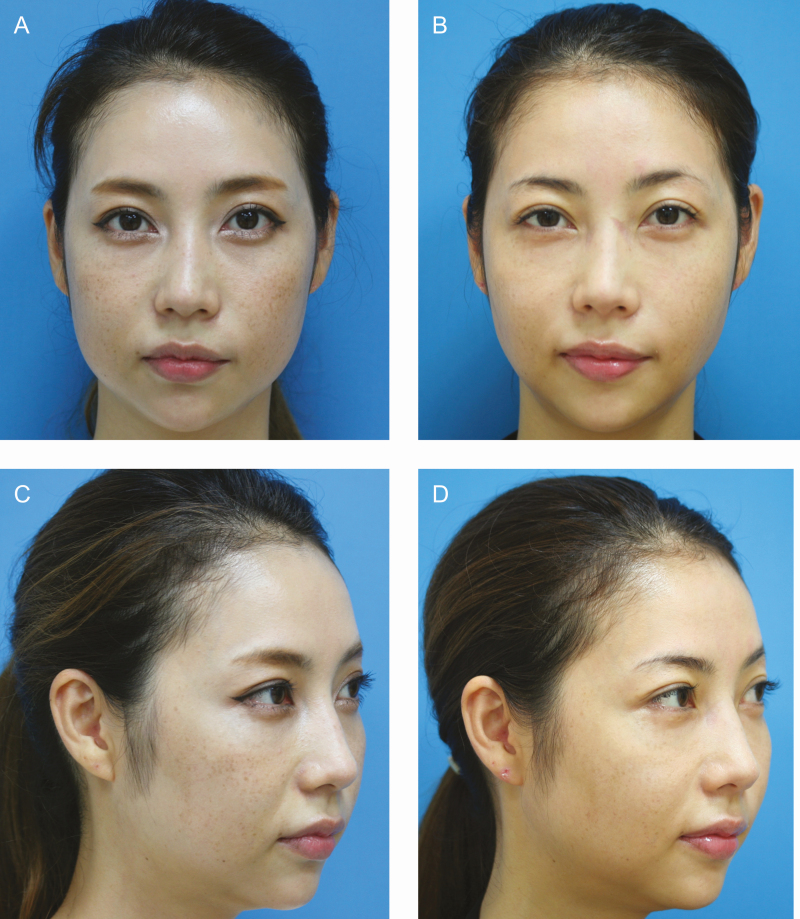
Patient 3: (A, C) Preoperative photographs of a 35-year-old female with open-fan-shaped cheeks were significantly improved after the buccal fat pad excision. (B, D) Twelve-month postoperative pictures were taken on September 29, 2017.

One-week postoperative observation showed moderate hematoma mass (83%) on the pocket space created after BFP excision in almost all patients. One patient had prolonged internal bleeding (3.3%) in an area extending from the lower eyelid down to the jaw and the cervical region. Eventually, the internal bleeding resolved spontaneously within 3 weeks postoperatively. There was no need for hematoma evacuations as all hematomas were minimal and resolved spontaneously. One patient was given a hyaluronic acid injection 6 months postoperatively to improve a minor dent (3.3%) that appeared on the upper cheek. This patient had a previous facial liposuction. The patient showed a steady recovery after the injection, and no additional treatment was provided. There were no major complications other than that. To address postoperative pain, pain medication was prescribed for the patients to take at home if needed. However, only 2 patients (6.7%) were found to have taken it postoperatively. There were no cases of infection. Three typical patients who underwent BFP excision surgery are shown in Figures 4-6.

## DISCUSSION

The target of this study was selected for the middle-age range who have copious or ptotic faces with the principal focus on female patients (28) as there were only 2 male patients. Although the data were not broad enough to evaluate the gender difference of BFP excision, it is possible that male patients may gain greater results from this surgery due to the thicker and larger BFP contributing to a ptotic face. This suggests that when BFP excision is well managed in male patients, the results can be more obvious than in female patients.

The Asian population was exclusively chosen in this study, and they appear to have relatively larger BFP and thicker skin than other ethnicities such as Caucasoid. Therefore, patients with an Asian background are ideal candidates for BFP excision with BFP potentially responsible for their greater ptotic faces. It is also noted that this contributes to safer removal of the BFP as thicker skin holds well after the excision.

To verify the efficacy of BFP excision for those patients, I examined if there is a correlation between the amount of an individual’s BFP and its contractionary effect on the lower face. In addition, both bilateral volumes and horizontal length of the lower face were measured preoperatively and postoperatively using 3D picture analysis devices. Although most previous studies measured BFP volume using ultrasound systems preoperatively and compared it with facial volumes,^11^ it was impossible to measure preoperative BFP volume with the Morpheus system, so the BFP weight gained from surgery was substituted for BFP volume. The results showed that both bilateral lower facial volumes and horizontal length became smaller and shorter postoperatively, and these differences were proven to be statistically significant. And there was no statistically significant difference between preoperative and 12-month postoperative body weight. These results strongly suggest that BFP volume may be responsible for voluminous lower face, namely if BFP excision is well performed, this surgery could be beneficial for the copious or ptotic face. It was also noted that there were no statistical correlations between the extirpated BFP weight and both bilateral lower facial volumes and horizontal length. Also, there was no significant difference in bilateral extirpated BFP weight. Those results support the hypothesis that the extirpated BFP weight does not have any correlation with lower facial volume and horizontal length, because the face is structured three-dimensionally by the musculoskeletal components. Perhaps the size reduction effect of the lower face does not depend on only the extirpated BFP weight, but rather this effect might also be gained from 3D soft tissue contracture surrounding the excised BFP. When scrutinizing the limitations of this study, preoperative and postoperative differences were objectively compared using lower facial volume and horizontal length. Measurement errors can occur when facial landmarks are imprecise or uneven due to slight differences in the position of each patient when recording the 3D picture. Hence, these data can be only used for reference, and it is not possible to conclude the value of this study by observing those facial scales only.

The surgical approach should be selected with respect to the 2 major methods. The Matarasso approach is located below the parotid duct opening, whereas the Stuzin approach is behind it as illustrated in Figure 3. It should be noted, however, that the size of the parotid duct’s opening varies depending on an individual’s anatomy, and in some rare cases it is not always easy to identify. In such cases, BFP should be approached from the oral vestibule facing the upper second molar for the Matarasso approach. For BFP excision for ptotic cheek in the elderly population, the Matarasso approach is more favorable as the distal end of the extension segment of BFP is easily accessed. If the BFP pocket is accessed from the Stuzin approach, the upper portion of the BFP extension or body part is the first tissue encountered. The body part of BFP (proximal region) should be resected conservatively after the BFP extension (distal region) is resected first, in order to avoid over-resection of the BFP. So that the Matarasso approach is the preferred method for the elderly population, as sagging of BFP extension is directly responsible for the ptotic lower face of this group. Extreme caution should be exercised when removing BFP located in a deeper area from the oral surface which is close to the skin surface to avoid over-resection. It is not an exaggeration to note that the success of this surgery depends on precise management of the BFP excision. However, it is not easy to clarify how the appropriate amount of BFP excision is decided. One clue is if the BFP spills out by itself after the opening of the BFP capsule, it can be excised without hesitation. When the remaining BFP starts retracting, this is the indication to stop the resection or just trim off only a little discreetly. This judgment could be the key to achieving a good result, and it should be learned from repeated experience of this surgery.

There are some difficult cases where BFP excisions may be encountered, such as patients with a history of mesotherapy injections,^12^ cheek liposuction, fat grafting on cheek, and facelift surgery. As a result of such treatments, their BFP may have degenerated, lost elasticity and consistency, and become difficult to excise. Accessing BFP can also be challenging in patients whose faces have greater depth as it is hard to reach the BFP pocket located deep inside from the mucosal entry. There are some cases in which a substantial portion of the BFP extension is herniated into the superficial layer of the face either congenitally or due to aging, whereas accessing the BFP lying deep in the lower cheek area from a small and narrow space on the oral mucosa can often be a painstaking task. This invaginated BFP into the cheek pocket was designated as Pseudo-herniation^13^ of the BFP. This phenomenon was observed, which is shown in Figure 4. 

If BFP excisions are made carelessly and too much is removed in patients with slimmer faces, there is the danger of a postoperative gaunt look. Careful evaluation of the face and conservative BFP excision for patients predisposed to the gaunt look is imperative to determine the viability of surgery. Other potential risks associated with BFP excision are injury to the mandibular nerve and the facial nerve. This type of nerve damage might be caused by blind or rough dissection extending over the BFP space. To avoid such injuries, BFP dissection should be always carried out visually and moderately.

**Figure 7.**
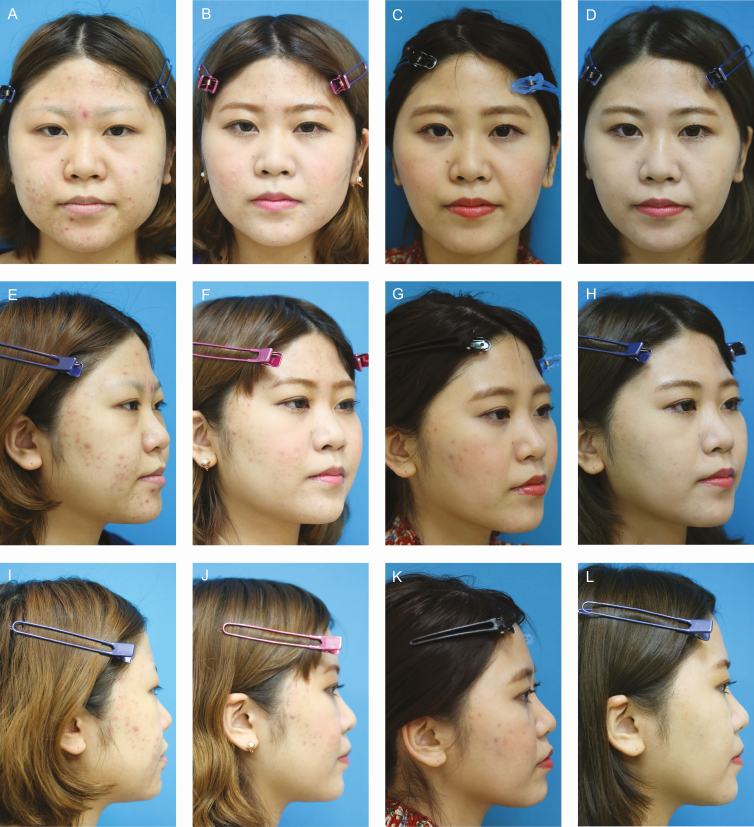
Patient 4: This 25-year-old female was selected separately from this study to see the long-term outcome of buccal fat pad (BFP) excision. BFP excision and transconjunctival eye bag surgery were performed simultaneously. This was one of the few patients whom the author succeeded in monitoring for 3 years postoperatively. Although BFP excision seems to play an important role in her facial slimming, body weight decrease during 3 years from 60 to 56 kg might partially contribute to it. (A, E, I) Preoperative, (B, F, J) 12 months, (C, G, K) 24 months, and (D, H, L) 36 months postoperative photographs are shown, and the last pictures were taken on June 13, 2019.

Bleeding prevention is essential to the success of this surgery. If bleeding occurs in the middle of surgery, precise BFP excision becomes extremely difficult due to poor vision of the surgical area, which can lead to unsatisfactory results. An incomplete hemostasis after the surgery may also cause prolonged swelling or longer postoperative recovery and associated downtime. However, if BFP excision is completed without any bleeding, the postoperative swelling is minimal, and the volume reduction and lifting effect of the lower face can be observed right after the operation. In patients examined 1 month postoperatively, obvious slimming of the cheek was observed in most of the patients. Greater results were usually gained over time. This is probably due to the prolonged healing process characteristic of BFP excision in which a fair amount of fat mass is resected at deep core in the face. Although some patients were dissatisfied after a 12-month period (6.6%) because the result was not as effective as they expected before the surgery, those patients should be told to wait for better results in the long term because the final optimal result might be gained after a long period of time as shown in Figure 7.

BFP excision is acknowledged as an effective surgery to improve the facial silhouette and capacious cheeks in younger patients. There were no specific studies performed to determine if it can also improve the ptotic face in middle-aged patients, with limited efficacy of BFP excision for this age group due to already reduced skin elasticity potentially leading to postoperative drooping. Therefore, BFP excision was carried out in this age group to observe the efficacy. When results were examined 12 months postoperatively, the observations were reasonable lower facial slimming, improvement of marionette lines, nasolabial grooves, and slight elevation of lateral mouth corner angles. These effects are thought to be obtained from the release of BFP weight burden at the lower face. While the improvements gained from BFP excision are not superior to facelift surgery, BFP excision can be a preliminary procedure before facelift surgery is seriously considered. However, BFP excision should not be performed in patients with severely diminished skin elasticity as excision of BFP volume may cause additional ptosis. Hence, careful selection of suitable candidates with good skin elasticity is also critical.

## CONCLUSIONS

In conclusion, comprehensive BFP excision procedures and details are explained in this study, and a sophisticated BFP excision is proven to have a safe and natural result for middle-aged patients. It must be noted, however, that this study was conducted only to relatively small number of patients in a limited time so that additional research should be executed with increased patient numbers for a longer time period. And, further follow-up in even older patients should be also carried out to verify its true effectiveness.

BFP excision appears to be an effective surgery for ptotic face in the middle age class. Objective improvements of facial scales gained from postoperative 3D facial pictures also support its effectiveness. Although BFP excision seems to be a simple and straightforward technique, there are critical rules to follow avoiding inadvertent side effects such as prolonged swelling or the infamous appearance of the gaunt look.

## References

[1] Branham GH. Lower eyelid blepharoplasty. Facial Plast Surg Clin North Am. 2016;24(2):129-138. doi: 10.1016/j.fsc.2015.12.004 27105798

[2] John JC. Periorbital surgery: forehead, brow, and midface. Facial Plast Surg Clin North Am. 2016;24(2):107-117.doi: 10.1016/j.fsc.2015.12.003 27105796

[3] Suh DH, Choi JH, Lee SJ, et al. Comparative histometric analysis of the effects of high-intensity focused ultrasound and radiofrequency on skin. J Cosmet Laser Ther. 2015;17(5):230-236. doi: 10.3109/14764172.2015.1022189 25723905

[4] Tavares JP, Oliveira CACP, Torres RP, et al. Facial thread lifting with suture suspension. Braz J Otorhinolaryngol. 2017;83(6):712-719. doi: 10.1016/j.bjorl.2017.03.015 28549872 PMC9449186

[5] Charafeddine AH, Drake R, McBride J, et al. Facelift: history and anatomy. Clin Past Surg. 2019;46(4):505-513. doi: 10.1016/j.cps.2019.05.001 31514803

[6] Chaffoo RA. Complications in facelift surgery: avoidance and management. Facial Plast Surg Clin North Am. 2013;21(4):551-558. doi: 10.1016/j.fsc.2013.07.007 24200374

[7] Cristel RT, Irvine LE. Common complications in rhytidectomy. Facial Plast Surg Clin North Am. 2019;27(4):519-527. doi: 10.1016/j.fsc.2019.07.008 31587771

[8] Stuzin JM, Wagstrom L, Kawamoto HK, et al. The anatomy and clinical applications of the buccal fat pad. Plast Reconstr Surg. 1990;85(1):29-37. doi: 10.1097/00006534-199001000-00006 2293733

[9] Tideman H, Bosanquet A, Scott J. Use of the buccal fat pad as a pedicled graft. J Oral Maxillofac Surg. 1986;44(6):435-440. doi: 10.1016/s0278-2391(86)80007-6 3457926

[10] Matarasso A. Buccal fad pad excision: aesthetic improvement of the midface. Ann Plast Surg. 1991;26(5):413-418. doi: 10.1097/00000637-199105000-00001 1952712

[11] Billur S, Sedat T, Medine B, et al. The excision of the buccal fat pad for cheek refinement: volumetric considerations. Aesthet Surg J. 2019;39(6):585-592. doi: 10.1093/asj/sjy188 30084868

[12] Plachouri MK, Georgiou S. Mesotherapy: safety profile and management of complications. J Cosmet Dermatol. 2019;18(6):1601-1605. doi: 10.1111/jocd.13115 31444843

[13] Matarasso A. Pseudoherniation of the buccal fat pad: a new clinical syndrome. Plast Reconstr Surg. 1997;100(3):723-730; discussion 731. doi: 10.1097/00006534-199709000-00030 9283575

